# Supplementary material for: Cropping system diversification for food production in Mindanao rubber plantations: a rice cultivar mixture and rice intercropped with mungbean
Source: PeerJ. 2017 Feb 8;5:e2975. doi: 10.7717/peerj.2975 (PMC5301974; doi:10.7717/peerj.2975)
Supplement: Supplemental Information 1 [file peerj-05-2975-s001.doc]

**Supplementary Figures and Tables**

**Supplementary Figures**

**Figure S1a.** The 2006 experimental design in the study of rice and mungbean cropping systems in rubber plantation intercropping. Rubber age was applied at the whole plot level, a farm, with three distinct farm replicates for each age. In each farm, there were three complete blocks in which subplots received each of the cropping system treatments (Table 1).

Rubber.age

1 year

3 years

farm1

farm 2

farm 3

farm 4

farm 5

farm 6

Block1

Block2

Block3

Block1

Block2

Block3

Block1

Block2

Block3

Block1

Block2

Block3

Block1

Block2

Block3

Block1

Block2

Block3

**Figure S1b.** A schematic drawing of treatment arrangements in each block. The intercropping treatments 1 to 8 in Table 1 were randomized and applied to subplots in the blocks.

**
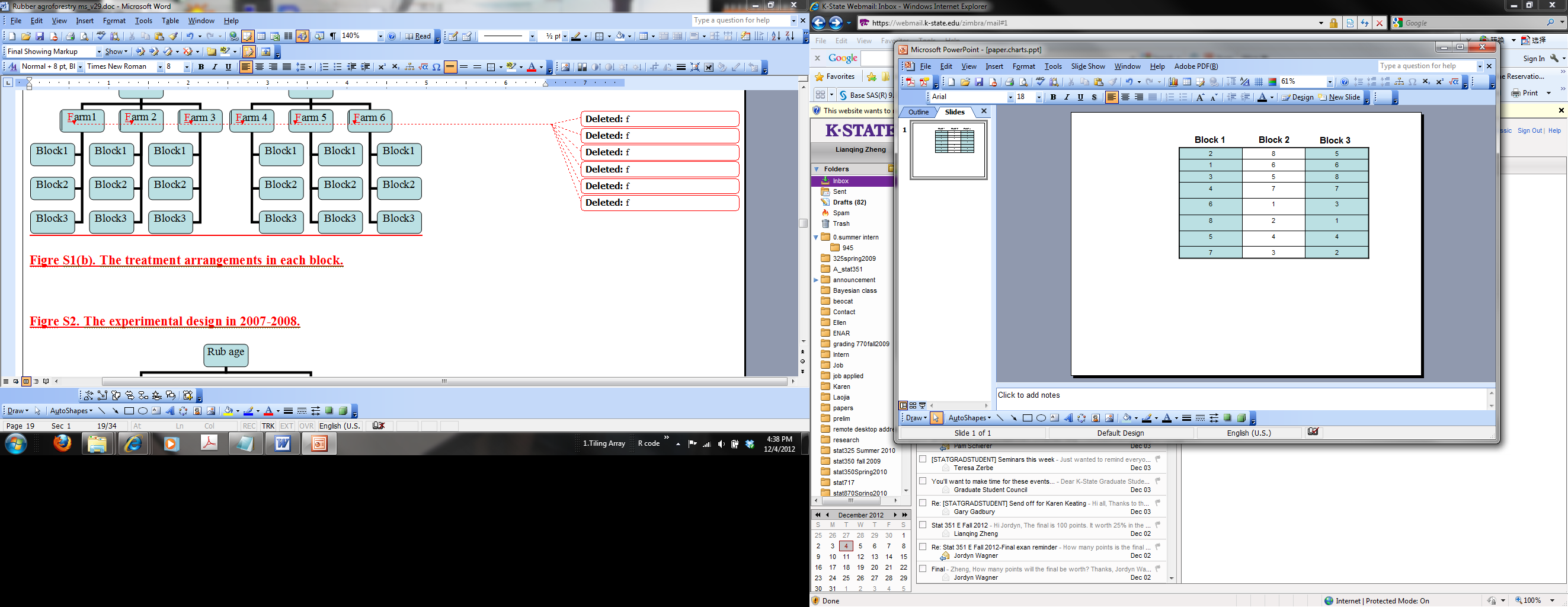
**

**Figure S2.** The 2007-2008 experimental design in the study of rice and mungbean cropping systems in rubber plantation intercropping. Farms were again whole plots, where each farm was studied for two years. The rubber tree age was different at each farm: 1, 1.5, 2, 2, 2.5 and 3 years. Since there was no replication of age group, age and farm do not appear in this design at the same time. The intercropping treatment arrangements in Table 1 were randomly applied in complete blocks as in 2006.

Year

2007

2008

farm1

age: 1

farm 2

age: 1.5

farm 3

age: 2

farm 1

age: 2

farm 2

age: 2.5

farm 3

age:3

Block1

Block2

Block3

Block1

Block2

Block3

Block1

Block2

Block3

Block1

Block2

Block3

Block1

Block2

Block3

Block1

Block2

Block3

**Figure S3.** 95% confidence intervals for the mean yields of Dinorado, UPL Ri-5 and mungbean in 2006 - 2008. Treatment abbreviations are given in Table 1.

**Figure S4.** 95% confidence interval for the land equivalent ration (LER). Treatment abbreviations are given in Table 1.

**Figure S5.** The relation between mean yields and ranks of yield in Dinorado treatment. The mean yield is the average yield per row for each site over seven treatments.

**Supplementary Tables**

**Table S1.** Planting and harvest dates for mungbean and rice cultivars UPL Ri-5 and Dinorado in experiments evaluating food crop production in young rubber plantations in Mindanao.

| **Year** | **Rubber tree age** | **Planting Dates and sites** | | **Crops and Harvest Dates** | |
| --- | --- | --- | --- | --- | --- |
|  | 1-year |  |  | UPL Ri-5 | Oct.10 |
|  |  | May 27 | Antipas | Dinorado | Oct.2 |
|  |  |  |  | Mungbean | Aug.15 – Sept.15 |
|  |  |  |  | UPL Ri-5 | Oct.15 |
|  |  | June 1 | Arakan | Dinorado | Oct.6 |
| **2006** |  |  |  | Mungbean | Aug.19 – Sept.19 |
|  |  |  |  | UPL Ri-5 | Oct.7 |
|  |  | May 25 | President Roxas | Dinorado | Sept.30 |
|  |  |  |  | Mungbean | Aug.12 – Sept.12 |
|  |  |  |  | UPL Ri-5 | Oct 12 |
|  |  | May 29 | Antipas | Dinorado | Oct 4 |
|  |  |  |  | Mungbean | Aug 17 – Sept 17 |
|  | 3-year |  |  | UPL Ri-5 | Ovt 16 |
|  |  | June 2 | Arakan | Dinorado | Oct 7 |
|  |  |  |  | Mungbean | Aug 20 – Sept 20 |
|  |  | May 26 |  | UPL Ri-5 | Oct 8 |
|  |  |  | President Roxas | Dinorado | Oct 1 |
|  |  |  |  | Mungbean | Aug 14 – Sept 14 |
| **2007** | >1 - 2 years |  |  | UPL Ri-5 | Sept.30 |
|  |  | May 16 | Naje, Arakan | Dinorado | Sept.23 |
|  |  |  |  | Mungbean | Aug.4 – Sept.4 |
|  |  |  |  | UPL Ri-5 | Oct.24 |
|  |  | June 12 | Poblacion, Arakan | Dinorado | Oct.15 |
|  |  |  |  | Mungbean | Aug.30 – Sept.30 |
|  |  |  |  | UPL Ri-5 | Nov.28 |
|  |  | July 17 | Doroluman, Arakan | Dinorado | Nov.20 |
|  |  |  |  | Mungbean | Oct.1 – Oct.30 |
| **2008** | >2- 3 year |  |  | UPL Ri-5 | Sept.5 |
|  |  | April 21 | Naje, Arakan | Dinorado | Aug.29 |
|  |  |  |  | Mungbean | Jul.10 – Aug.10 |
|  |  |  | Sitio Sabang, | UPL Ri-5 | Sept.1 |
|  |  | April 19 | Poblacion, Arakan | Dinorado | Aug.24 |
|  |  |  |  | Mungbean | Jul.8 – Aug.8 |
|  |  |  |  | UPL Ri-5 | Sept 6 |
|  |  | April 22 | Doroluman, Arakan | Dinorado | Aug 30 |
|  |  |  |  | Mungbean | July 12 – Aug 12 |

**Table S2:** Analysis of variance table for 2006 studies of the effects of rubber tree age and cropping treatments. The source column includes all the treatments in this design. The second column indicates whether the treatments are random effects or fixed effects. The mixed effect linear models were applied to the 2006 experiment since the Farms and the Blocks are random effects and rubber tree age is a fixed effect. Each crop (Dinorado, UPL Ri-5 and mungbean) did not appear in each of the eight intercropping treatments (Table 1). Therefore, the analyses were performed for the three crops separately (Columns: Dinorado model, UPL model and mungbean model).

| **Source** | **Random**  **Effect** | **df (All CT)** | **df (Dinorado model)** | **df (UPL model)** | **df(Mungbean model)** |
| --- | --- | --- | --- | --- | --- |
| **RA**  **F(RA)**  **B(F RA)** | Random  Random | 1  2(3-1) = 4  2(3)(3-1)=12 | 1  2(3-1) = 4  2(3)(3-1)=12 | 1  2(3-1) = 4  2(3)(3-1)=12 | 1  2(3-1) = 4  2(3)(3-1)=12 |
| **CT**  **CT*RA**  **CT*F(RA)**  **CT*B(F RA)** | Random  Random | 8-1 = 7  7 = 7  4(7)=28  84 | 5-1=4  1(4)=4  4(4)=16  4(12)=48 | 5-1=4  1(4)=4  4(4)=16  4(12)=48 | 4-1=3  3  12  36 |
| Total |  | 144 | 90 | 90 | 72 |

Where RA stands for the 2 rubber tree age. F(RA) stands for the 3 farms per RA. B(F RA) stands for 3 blocks per farm. CT is the 8 intercropping treatments.

**Table S3.** Analysis of Variance Table for 2007-2008.

| **Source** | **Random**  **Effect** | **df (All CT)** | **df (Dinorado model)** | **df (UPL model)** | **df(MungBean model)** |
| --- | --- | --- | --- | --- | --- |
| **F**  **B(F)** | Random  Random | 3-1 = 2  3(3-1)=6 | 3-1 = 2  3(3-1)=6 | 3-1 = 2  3(3-1)=6 | 3-1 = 2  3(3-1)=6 |
| **CT**  **CT*F**  **CT*B(F)** | Random  Random | 8-1 = 7  14  42 | 5-1 = 4  8  24 | 5-1 = 4  8  24 | 4-1 = 3  6  18 |
| **Y**  **Y*F**  **Y*B(F)**  **Y*CT**  **Y*CT*F**  **Y*CT*B(F)** | Random  Random  Random  Random | 2-1 = 1  2  3(3-1)=6  8-1 = 7  14  42 | 2-1 = 1  2  6  4  8  24 | 2-1 = 1  2  6  4  8  24 | 2-1 = 1  2  6  3  6  18 |
| Total |  | 144 | 90 | 90 | 72 |

Where F stands for the 3 farms. B(F) stands for 3 blocks per farm. CT is the 8 intercropping treatments. Y stands for the year effect.

**Table S4. 95% confidence intervals for Figure S1. Treatment abbreviations are given in Table 1.**

| **Crops** | **Treatment** | **year** | **Mean** | **Standard error** | **Lower limit** | **Upper limit** |
| --- | --- | --- | --- | --- | --- | --- |
| **Dinorado** | Monoculture | 2006 | 486.625 | 96.759 | 296.9776 | 676.2721 |
|  | Monoculture | 2007 | 626.035 | 142.313 | 347.1011 | 904.9695 |
|  | Monoculture | 2008 | 768.449 | 71.155 | 628.9851 | 907.9132 |
|  | RM | 2006 | 524.034 | 103.183 | 321.7957 | 726.2717 |
|  | RM | 2007 | 577.109 | 147.23 | 288.539 | 865.6793 |
|  | RM | 2008 | 728.347 | 94.74 | 542.6575 | 914.0364 |
|  | 0.2MB | 2006 | 470.381 | 103.586 | 267.3521 | 673.4094 |
|  | 0.2MB | 2007 | 561.47 | 138.306 | 290.3897 | 832.5507 |
|  | 0.2MB | 2008 | 725.384 | 99.157 | 531.0365 | 919.7309 |
|  | 0.5MB | 2006 | 625.593 | 101.311 | 427.0228 | 824.1639 |
|  | 0.5MB | 2007 | 1082.905 | 592.883 | -79.1463 | 2244.956 |
|  | 0.5MB | 2008 | 1066.368 | 157.119 | 758.415 | 1374.322 |
|  | 0.8MB | 2006 | 761.669 | 145.495 | 476.4983 | 1046.841 |
|  | 0.8MB | 2007 | 648.066 | 186.12 | 283.271 | 1012.862 |
|  | 0.8MB | 2008 | 1108.191 | 167.801 | 779.3001 | 1437.081 |
| **UPL Ri-5** | Monoculture | 2006 | 651.044 | 116.843 | 422.031 | 880.0568 |
|  | Monoculture | 2007 | 1054.351 | 192.827 | 676.4104 | 1432.292 |
|  | Monoculture | 2008 | 1052.034 | 86.346 | 882.7964 | 1221.272 |
|  | RM | 2006 | 604.86 | 97.566 | 413.6304 | 796.0902 |
|  | RM | 2007 | 1027.33 | 194.223 | 646.6534 | 1408.006 |
|  | RM | 2008 | 827.741 | 43.035 | 743.3928 | 912.09 |
|  | 0.2MB | 2006 | 668.22 | 101.898 | 468.5001 | 867.94 |
|  | 0.2MB | 2007 | 1154.098 | 252.443 | 659.3098 | 1648.886 |
|  | 0.2MB | 2008 | 975.49 | 72.434 | 833.5182 | 1117.461 |
|  | 0.5MB | 2006 | 790.037 | 121.092 | 552.6969 | 1027.376 |
|  | 0.5MB | 2007 | 977.268 | 234.28 | 518.0803 | 1436.456 |
|  | 0.5MB | 2008 | 1111.432 | 67.378 | 979.3713 | 1243.493 |
|  | 0.8MB | 2006 | 928.471 | 171.119 | 593.0778 | 1263.864 |
|  | 0.8MB | 2007 | 1029.449 | 306.268 | 429.1628 | 1629.735 |
|  | 0.8MB | 2008 | 1475.408 | 137.544 | 1205.822 | 1744.993 |
| **Mung bean** | Monoculture | 2006 | 187 | 30.666 | 126.8945 | 247.1055 |
|  | Monoculture | 2007 | 321.856 | 34.667 | 253.9075 | 389.8036 |
|  | Monoculture | 2008 | 412.222 | 60.731 | 293.1892 | 531.2553 |
|  | 0.2MB | 2006 | 275.967 | 57.008 | 164.2307 | 387.7026 |
|  | 0.2MB | 2007 | 496 | 45.317 | 407.1792 | 584.8208 |
|  | 0.2MB | 2008 | 363.889 | 29.788 | 305.5037 | 422.2741 |
|  | 0.5MB | 2006 | 219.107 | 30.161 | 159.9902 | 278.2231 |
|  | 0.5MB | 2007 | 429.733 | 31.046 | 368.8827 | 490.584 |
|  | 0.5MB | 2008 | 363.333 | 27.386 | 309.6565 | 417.0101 |
|  | 0.8MB | 2006 | 173.192 | 28.785 | 116.7733 | 229.61 |
|  | 0.8MB | 2007 | 356.292 | 34.347 | 288.9725 | 423.6108 |
|  | 0.8MB | 2008 | 338.889 | 18.801 | 302.0382 | 375.7396 |

**Table S5. 95% confidence intervals for land equivalent ratio (LER). Treatment abbreviations are given in Table 1.**

| **Treatment** | **Year** | **Mean** | **SE** | **LL** | **UL** |
| --- | --- | --- | --- | --- | --- |
| **RM** | 2006 | 1.528 | 0.218 | 1.100407 | 1.955507 |
| **RM** | 2007 | 0.982 | 0.076 | 0.834135 | 1.130342 |
| **RM** | 2008 | 0.87 | 0.054 | 0.764536 | 0.974999 |
| **0.2MB** | 2006 | 1.23 | 0.125 | 0.886445 | 1.377868 |
| **0.2MB** | 2007 | 1.297 | 0.167 | 1.017491 | 1.670536 |
| **0.2MB** | 2008 | 0.965 | 0.078 | 0.930676 | 1.235386 |
| **0.5MB** | 2006 | 1.132 | 0.092 | 0.808567 | 1.169465 |
| **0.5MB** | 2007 | 1.344 | 0.158 | 0.932113 | 1.549883 |
| **0.5MB** | 2008 | 1.083 | 0.07 | 0.861242 | 1.137091 |
| **0.8MB** | 2006 | 0.989 | 0.121 | 0.993952 | 1.466434 |
| **0.8MB** | 2007 | 1.241 | 0.203 | 0.899472 | 1.695471 |
| **0.8MB** | 2008 | 0.999 | 0.08 | 0.807798 | 1.121289 |

**Table S6.** Mean yield per row for each treatment in each site in year 2006 and 2007-2008. Treatment abbreviations are given in Table 1.

|  | 2006 site1 | 2006 site2 | 2006 site3 | 2006 site4 | 2006 site5 | 2006 site6 | 2007 site1 | 2007 site2 | 2007 site3 | 2008 site1 | 2008 site2 | 2008 site3 |
| --- | --- | --- | --- | --- | --- | --- | --- | --- | --- | --- | --- | --- |
| Dinorado | 1237.66 | 640.80 | 118.87 | 216.20 | 412.72 | 293.50 | 817.86 | 868.29 | 191.96 | 974.55 | 526.51 | 804.28 |
| UPL | 1597.92 | 649.84 | 134.79 | 390.48 | 503.01 | 630.22 | 1705.01 | 1027.70 | 430.34 | 1191.54 | 827.27 | 1137.30 |
| RM | 1333.35 | 573.96 | 170.13 | 472.41 | 391.21 | 494.12 | 1219.73 | 1063.39 | 258.60 | 974.02 | 630.58 | 759.35 |
| 0.5 MB | 799.00 | 547.37 | 255.36 | 236.13 | 487.66 | 395.02 | 1123.15 | 607.94 | 442.80 | 771.34 | 626.22 | 787.54 |
| 0.8 MB | 456.23 | 195.62 | 313.88 | 155.81 | 335.07 | 250.24 | 439.42 | 558.56 | 360.37 | 590.33 | 493.63 | 504.46 |
| 0.2 MB | 1096.43 | 460.28 | 198.23 | 372.05 | 447.95 | 433.68 | 966.77 | 1051.70 | 337.81 | 905.08 | 618.15 | 736.15 |
| MB | 76.67 | 85.00 | 330.00 | 0.00 | 280.00 | 163.33 | 266.67 | 321.67 | 377.23 | 340.00 | 323.33 | 573.33 |
| Mean | 942.47 | 450.41 | 217.32 | 263.30 | 408.23 | 380.02 | 934.09 | 785.61 | 342.73 | 820.98 | 577.96 | 757.49 |

**Table S7.** The ranks of the mean yield per row for each treatment in each site in year 2006 and 2007-2008. Treatment abbreviations are given in Table 1. The mean yield per row for each treatment in Table S5 are ranked for each site in each column.

|  | 2006 site1 | 2006 site2 | 2006 site3 | 2006 site4 | 2006 site5 | 2006 site6 | 2007 site1 | 2007 site2 | 2007 site3 | 2008 site1 | 2008 site2 | 2008 site3 | Mean Rank |
| --- | --- | --- | --- | --- | --- | --- | --- | --- | --- | --- | --- | --- | --- |
| Dinorado | 5 | 6 | 1 | 3 | 4 | 3 | 3 | 4 | 1 | 6 | 3 | 6 | 3.75 |
| UPL | 7 | 7 | 2 | 6 | 7 | 7 | 7 | 5 | 6 | 7 | 7 | 7 | 6.25 |
| RM | 6 | 5 | 3 | 7 | 3 | 6 | 6 | 7 | 2 | 5 | 6 | 4 | 5.00 |
| 0.5 MB | 3 | 4 | 5 | 4 | 6 | 4 | 5 | 3 | 7 | 3 | 5 | 5 | 4.50 |
| 0.8 MB | 2 | 2 | 6 | 2 | 2 | 2 | 2 | 2 | 4 | 2 | 2 | 1 | 2.42 |
| 0.2 MB | 4 | 3 | 4 | 5 | 5 | 5 | 4 | 6 | 3 | 4 | 4 | 3 | 4.17 |
| MB | 1 | 1 | 7 | 1 | 1 | 1 | 1 | 1 | 5 | 1 | 1 | 2 | 1.92 |

**Table S8.** Economic value for each treatment in each site in year 2006 and 2007-2008. Treatment abbreviations are given in Table 1. The mean economic value for each treatment is based on the mean yield per row (Table S5) according to the economic value weight 1.4 : 1 (Dinorado : UPL Ri-5) and 1.4 : 1 (Mungbean : UPL Ri-5).

|  | 2006 site1 | 2006 site2 | 2006 site3 | 2006 site4 | 2006 site5 | 2006 site6 | 2007 site1 | 2007 site2 | 2007 site3 | 2008 site1 | 2008 site2 | 2008 site3 |
| --- | --- | --- | --- | --- | --- | --- | --- | --- | --- | --- | --- | --- |
| Dinorado | 1732.73 | 897.11 | 166.42 | 302.68 | 577.81 | 410.90 | 1145.00 | 1215.61 | 268.74 | 1364.37 | 737.12 | 1126.00 |
| UPL | 1597.92 | 649.84 | 134.79 | 390.48 | 503.01 | 630.22 | 1705.01 | 1027.70 | 430.34 | 1191.54 | 827.27 | 1137.30 |
| RM | 1553.68 | 646.31 | 218.00 | 518.65 | 431.26 | 570.36 | 1328.32 | 1205.43 | 284.99 | 1142.54 | 708.46 | 862.56 |
| 0.5 MB | 907.73 | 674.01 | 333.02 | 254.90 | 586.75 | 483.52 | 1366.81 | 764.00 | 560.81 | 945.49 | 747.65 | 965.89 |
| 0.8 MB | 549.88 | 247.19 | 426.91 | 178.59 | 440.07 | 324.12 | 568.11 | 717.19 | 492.87 | 754.57 | 629.42 | 662.74 |
| 0.2 MB | 1304.49 | 553.74 | 252.35 | 406.82 | 540.94 | 512.22 | 1075.45 | 1259.09 | 410.28 | 1083.04 | 721.01 | 890.85 |
| MB | 107.33 | 119.00 | 462.00 | 0.00 | 392.00 | 228.67 | 373.33 | 450.33 | 528.13 | 476.00 | 452.67 | 802.67 |
| Mean | 1107.68 | 541.03 | 284.78 | 293.16 | 495.98 | 451.43 | 1080.29 | 948.48 | 425.17 | 993.94 | 689.08 | 921.14 |

**Table S9.** The ranks of the economic value in each treatment in each site in year 2006 and 2007-2008. Treatment abbreviations are given in Table 1. The treatment economic values are ranked according to Table S7 for each sites in each column.

|  | 2006 site1 | 2006 site2 | 2006 site3 | 2006 site4 | 2006 site5 | 2006 site6 | 2007 site1 | 2007 site2 | 2007 site3 | 2008 site1 | 2008 site2 | 2008 site3 | Mean Rank |
| --- | --- | --- | --- | --- | --- | --- | --- | --- | --- | --- | --- | --- | --- |
| Dinorado | 7 | 7 | 2 | 4 | 6 | 3 | 4 | 6 | 1 | 7 | 5 | 6 | 4.83 |
| UPL | 6 | 5 | 1 | 5 | 4 | 7 | 7 | 4 | 4 | 6 | 7 | 7 | 5.25 |
| RM | 5 | 4 | 3 | 7 | 2 | 6 | 5 | 5 | 2 | 5 | 3 | 3 | 4.17 |
| 0.5 MB | 3 | 6 | 5 | 3 | 7 | 4 | 6 | 3 | 7 | 3 | 6 | 5 | 4.83 |
| 0.8 MB | 2 | 2 | 6 | 2 | 3 | 2 | 2 | 2 | 5 | 2 | 2 | 1 | 2.58 |
| 0.2 MB | 4 | 3 | 4 | 6 | 5 | 5 | 3 | 7 | 3 | 4 | 4 | 4 | 4.33 |
| MB | 1 | 1 | 7 | 1 | 1 | 1 | 1 | 1 | 6 | 1 | 1 | 2 | 2.00 |

**Table S10.** Crop height and disease and pest severity (**2006**) in intercropping systems (Table 1) of mungbean and two rice cultivars (Dinorado and UPL Ri-5), in rubber plantations in Mindanao. Results of pair-wise comparisons are indicated by superscripts.

| Crops | Treatment | Crop Height | | Panicle Blast | | Brown Spot | | Brown leaf spot | | Rice Bug | |
| --- | --- | --- | --- | --- | --- | --- | --- | --- | --- | --- | --- |
|  |  | Mean | (SD) | Mean | (SD) | Mean | (SD) | Mean | (SD) | Mean | (SD) |
| Dinorado | Monoculture | 148.70^a^ | 5.08 | 1.39^a^ | 0.20 | 1.01^a^ | 0.09 | 1.26^a^ | 0.07 | 2.17^ab^ | 1.06 |
|  | RM | 147.05^a^ | 5.08 | 1.33^a^ | 0.20 | 1.12^a^ | 0.09 | 1.26^a^ | 0.07 | 2.83^ab^ | 1.06 |
|  | 0.5MB | 133.27^b^ | 5.08 | 1.06^a^ | 0.20 | 1.11^a^ | 0.09 | 1.32^a^ | 0.07 | 1.94^b^ | 1.06 |
|  | 0.8MB | 142.31^ab^ | 5.08 | 1.44^a^ | 0.20 | 0.92^a^ | 0.09 | 1.23^a^ | 0.07 | 1.83^ab^ | 1.06 |
|  | 0.2MB | 144.26^ab^ | 5.08 | 1.72^a^ | 0.20 | 1.12^a^ | 0.09 | 1.29^a^ | 0.07 | 1.61^a^ | 1.06 |
| UPL Ri-5 | Monoculture | 115.11^a^ | 4.31 | 2.33^a^ | 0.21 | 0.83^a^ | 0.36 | 3.56^a^ | 0.21 | 5.28^a^ | 1.44 |
|  | RM | 117.33^a^ | 4.31 | 1.89^a^ | 0.21 | 1.22^a^ | 0.36 | 3.11^a^ | 0.21 | 4.61^a^ | 1.44 |
|  | 0.5MB | 112.55^a^ | 4.31 | 1.94^a^ | 0.21 | 0.89^a^ | 0.36 | 3.67^a^ | 0.21 | 4.94^a^ | 1.44 |
|  | 0.8MB | 112.15^a^ | 4.31 | 2.17^a^ | 0.21 | 1.22^a^ | 0.36 | 3.11^a^ | 0.21 | 4.94^a^ | 1.44 |
|  | 0.2MB | 112.92^a^ | 4.31 | 2.06^a^ | 0.21 | 1.06^a^ | 0.36 | 3.33^a^ | 0.21 | 4.83^a^ | 1.44 |
| Mungbean | Monoculture | 62.83^a^ | 12.27 | ─ | ─ | ─ | ─ | ─ | ─ | ─ | ─ |
|  | 0.5MB | 65.13^a^ | 12.27 | ─ | ─ | ─ | ─ | ─ | ─ | ─ | ─ |
|  | 0.8MB | 61.22^a^ | 12.27 | ─ | ─ | ─ | ─ | ─ | ─ | ─ | ─ |
|  | 0.2MB | 59.03^a^ | 12.27 | ─ | ─ | ─ | ─ | ─ | ─ | ─ | ─ |

The monoculture treatment refers to the crops in Dinarado, UPL Ri-5 and mungbean alone. Treatments RM, 0.5MB, 0.8MB and 0.2 MB refer to the intercropping treatment in Table 2. Superscripts a, b: if the means contain the same letters, then there is no significant difference in the pair-wise comparison. If the means contain different letters, then there is a significant difference at the 0.05 level.

**Table S11.** P-values from treatment effects on crop height and disease and pest severity (**2006**) in intercropping systems (Table 1) of mungbean and two rice cultivars (Dinorado and UPL Ri-5), in rubber plantations in Mindanao.

| Crops | Effects | Crop Height | Panicle Blast | Brown Spot | Brown leaf spot | Rice Bug |
| --- | --- | --- | --- | --- | --- | --- |
| Dinorado | Treatment | **0.01** | 0.20 | 0.31 | 0.77 | 0.06 |
|  | Rubber age | 0.69 | 0.76 | **0.03** | 0.76 | 0.57 |
|  | Trt*Rubber age | 0.08 | 0.63 | 0.39 | **0.042** | 0.67 |
| UPL Ri-5 | Treatment | 0.66 | 0.53 | 0.74 | 0.08 | 0.83 |
|  | Rubber age | 0.96 | **0.001** | 0.50 | 0.77 | 0.73 |
|  | Trt*Rubber age | 0.44 | 0.17 | 0.58 | 0.85 | 0.24 |
| Mungbean | Treatment | 0.48 | ─ | ─ | ─ | ─ |
|  | Rubber age | 0.30 | ─ | ─ | ─ | ─ |
|  | Trt*Rubber age | 0.94 | ─ | ─ | ─ | ─ |

Treatment stands for the 8 intercropping treatments (Table 2). Trt*Rubber age stand for the treatment and rubber age interaction. Bold p values are significant at the 0.05 level.

**Table S12.** Disease and pest severity (**2007-2008**) in intercropping systems (Table 1) of mungbean and two rice cultivars (Dinorado and UPL Ri-5), in rubber plantations in Mindanao. Results of pair-wise comparisons are indicated by superscripts.

| Crops | Treatment | Brown Spot | | Panicle Blast | | Leaf Blast | | Pod Rot | |
| --- | --- | --- | --- | --- | --- | --- | --- | --- | --- |
|  |  | Mean | (SD) | Mean | (SD) | Mean | (SD) | Mean | (SD) |
| Dinorado | Monoculture | 3.22 | 0.47 | 1.09 | 0.23 | 0.61 | 0.25 | ─ | ─ |
|  | RM | 2.33 | 0.47 | 1.23 | 0.32 | 0.78 | 0.25 | ─ | ─ |
|  | 0.5MB | 3.33 | 0.47 | 0.75 | 0.26 | 0.61 | 0.25 | ─ | ─ |
|  | 0.8MB | 3.50 | 0.47 | 1.24 | 0.26 | 0.50 | 0.25 | ─ | ─ |
|  | 0.2MB | 2.89 | 0.47 | 1.18 | 0.26 | 0.72 | 0.25 | ─ | ─ |
| UPL Ri-5 | Monoculture | 2.44 | 0.32 | 0.63 | 0.27 | 0.00 | 0.14 | ─ | ─ |
|  | RM | 2.00 | 0.32 | 0.10 | 0.27 | 0.14 | 0.08 | ─ | ─ |
|  | 0.5MB | 2.22 | 0.32 | 0.09 | 0.27 | 0.35 | 0.11 | ─ | ─ |
|  | 0.8MB | 2.17 | 0.32 | 0.52 | 0.20 | 0.35 | 0.12 | ─ | ─ |
|  | 0.2MB | 1.83 | 0.32 | 0.14 | 0.27 | 0.35 | 0.11 | ─ | ─ |
| Mungbean | Monoculture | ─ | ─ | ─ | ─ | ─ | ─ | 17.22 | 5.09 |
|  | 0.5MB | ─ | ─ | ─ | ─ | ─ | ─ | 10.56 | 5.09 |
|  | 0.8MB | ─ | ─ | ─ | ─ | ─ | ─ | 10.83 | 5.09 |
|  | 0.2MB | ─ | ─ | ─ | ─ | ─ | ─ | 29.61 | 5.09 |

There are no significant differences in the means in all pair-wise comparisons.

**Table S13.** P-values from treatment effects on disease and pest severity (**2007-2008**) in intercropping systems (Table 1) of mungbean and two rice cultivars (Dinorado and UPL Ri-5), in rubber plantations in Mindanao.

| Crops | Effects | Brown Spot | Panicle Blast | Leaf Blast | Pod Rot |
| --- | --- | --- | --- | --- | --- |
| Dinorado | Treatment | 0.18 | 0.49 | 0.78 | ─ |
|  | Year | 0.11 | 0.98 | 0.20 | ─ |
|  | Trt*Year | 0.38 | 0.96 | 0.07 | ─ |
| UPL Ri-5 | Treatment | 0.69 | 0.26 | 0.20 | ─ |
|  | Year | **0.02** | 0.74 | **0.0008** | ─ |
|  | Trt*Year | 0.31 | 0.34 | 0.20 | ─ |
| Mungbean | Treatment | ─ | ─ | ─ | **0.04** |
|  | Year | ─ | ─ | ─ | 0.08 |
|  | Trt*Year | ─ | ─ | ─ | 0.10 |

Bold p-values are significant at the 0.05 level.
