# Supplementary material for: Cropping system diversification for food production in Mindanao rubber plantations: a rice cultivar mixture and rice intercropped with mungbean
Source: PeerJ. 2017 Feb 8;5:e2975. doi: 10.7717/peerj.2975 (PMC5301974; doi:10.7717/peerj.2975)
Supplement: Supplemental Information 2 [file peerj-05-2975-s002.pdf]

SAS code and R scripts

1. Example SAS code for analyzing the yield data set in 2006 (L. Zheng and L. Murray)

```
OPTIONS NODATE pageno=1;
data one;
input age farm $ trt $ crop $ rep1yld rep2yld rep3yld @@;
LINES;
1	Alex	2d	d	1383.76	1093.73	1235.5
1	Alex	3u	u	1810	1600	1383.76
1	Alex	4d	d	1482.6	1660.22	988.4
1	Alex	4u	u	1548.493333	1274.266667	1089.833333
1	Alex	5d	d	1220.75	1074.25	1482.6
1	Alex	5u	u	1976.8	1449.653333	1845.133333
1	Alex	5m	m	30	40	50
1	Alex	6d	d	2316	1889.17	1768.5
1	Alex	6u	u	2767.52	2148.52	1747.26
1	Alex	6m	m	25	43.75	62.5
1	Alex	7d	d	1482.6	1220.75	1105.3125
1	Alex	7u	u	1750	1304.325	1267.7325
1	Alex	7m	m	60	50	75
1	Alex	8m	m	85	55	90
1	Edwin	2d	d	643.21	297.29	981.885
1	Edwin	3u	u	293.1	695.056	961.368
1	Edwin	4d	d	566.9375	240	549.5625
1	Edwin	4u	u	655.425	291.3	1018.746667
1	Edwin	5d	d	839.125	1198.75	536.065
1	Edwin	5u	u	867.4333333	776.8	663.5166667
1	Edwin	5m	m	280	230	360
1	Edwin	6d	d	642.2	503.48	271.88
1	Edwin	6u	u	926.4	683.9	390.8
1	Edwin	6m	m	193.75	106.25	6.25
1	Edwin	7d	d	728.25	50.9775	928.15
1	Edwin	7u	u	587.3875	424.8125	687.5
1	Edwin	7m	m	25	40	25
1	Edwin	8m	m	15	230	10
1	Rodolfo	2d	d	53.735	185.63	117.24
1	Rodolfo	3u	u	83.045	174.78	146.55
1	Rodolfo	4d	d	293.1	91.39	512.925
1	Rodolfo	4u	u	154.6916667	73.275	24.425
1	Rodolfo	5d	d	92.815	146.55	97.7
1	Rodolfo	5u	u	293.1	254.02	65.13333333
1	Rodolfo	5m	m	360	330	340
1	Rodolfo	6d	d	244.25	537.35	195.4
1	Rodolfo	6u	u	242.75	310.72	386
1	Rodolfo	6m	m	287.5	275	375
1	Rodolfo	7d	d	61.75	22.23	135.85
1	Rodolfo	7u	u	241.25	84.9625	145.65
1	Rodolfo	7m	m	400	490	700
1	Rodolfo	8m	m	385	285	320
3	Wilson	2d	d	137.137	236.873	274.593
3	Wilson	3u	u	507.832	308.1	355.5
3	Wilson	4d	d	524.9225	135.905	206.275
3	Wilson	4u	u	794.1666667	582.2166667	407.575
3	Wilson	5d	d	248.575	151.375	303.975
3	Wilson	5u	u	894.2333333	431.55	566.24
3	Wilson	5m	m	.	.	.
3	Wilson	6d	d	485.35	1025.43	197.68
3	Wilson	6u	u	627.25	1844.33	494.2
3	Wilson	6m	m	.	.	.
3	Wilson	7d	d	320.33	74.3225	257.4
3	Wilson	7u	u	1119.775	458.63	559.8875
3	Wilson	7m	m	.	.	.
3	Wilson	8m	m	.	.	.
3	Arsenia	2d	d	469.26	391.483	377.412
3	Arsenia	3u	u	536.777	526.219	446.034
3	Arsenia	4d	d	263.725	353.6725	133.47
3	Arsenia	4u	u	514.8	667.2116667	273.475
3	Arsenia	5d	d	427.11	576.105	671.24
```

```
3       Arsenia 5u      u       617.76  703.2666667     1078.236667
3       Arsenia 5m      m       245     291.6   280
3       Arsenia 6d      d       294.32  899.97  497.88
3       Arsenia 6u      u       637.06  1179.23 360.58
3       Arsenia 6m      m       226     291.625 255.25
3       Arsenia 7d      d       222.5325        326.155 495.0825
3       Arsenia 7u      u       600.3075        589.615 426.1725
3       Arsenia 7m      m       437.5   495.5   466.5
3       Arsenia 8m      m       262.5   309.2   268.3
3       Sim     2d      d       119.922 343.65  416.938
3       Sim     3u      u       428.85  795.97  665.85
3       Sim     4d      d       500.325 457.44  471.735
3       Sim     4u      u       543.4333333     397.0833333     577.0666667
3       Sim     5d      d       1014.36 524.15  655.185
3       Sim     5u      u       540.7   559.4166667     637.6666667
3       Sim     5m      m       150     160     140
3       Sim     6d      d       600.32  628.98  711.89
3       Sim     6u      u       383.6   767.2   815.16
3       Sim     6m      m       125     162.5   162.5
3       Sim     7d      d       398.16  286.77  350.2275
3       Sim     7u      u       603.125 721.3   455.525
3       Sim     7m      m       375     250     250
3       Sim     8m      m       150     200     140
;

PROC PRINT data=one;
 TITLE 'Yield 2006 DATA';
 RUN;

 data two; set one;
 rep = 1; yield =rep1yld; output;
 rep = 2; yield =rep2yld; output;
 rep = 3; yield =rep3yld; output;
 run;
proc print data=two;
 run;

/*Dinorado data*/
proc mixed data = two covtest cl;
 where crop = 'd';
 class age farm rep trt;
 model yield = trt age trt*age/ddfm=satterth outp=newd;
 random farm(age) rep(farm age) trt*farm(age);
 lsmeans age trt age*trt/cl;
 lsmeans trt/diff adjust=TUKEY;
 lsmeans age*trt/diff slice=age;
 run;

proc print data=newd;
 run;
 proc univariate normal plot data=newd;
 var resid;
 run;

/*UPL Ri-5 data*/
proc mixed data = two covtest cl;
 where crop = 'u';
 class age farm rep trt;
 model yield = trt age trt*age/ddfm=satterth outp=newu;
 random farm(age) rep(farm age) trt*farm(age);
 lsmeans age trt age*trt/cl;
 lsmeans trt/diff adjust=TUKEY;
 lsmeans age*trt/diff slice=age;
 run;

proc print data=newu;
 run;
proc univariate normal plot data=newu;
 var resid;
 run;
```

```
/*Mungbean data*/
proc mixed data = two covtest cl;
 where crop = 'm';
 class age farm rep trt;
 model yield = trt age trt*age/ddfm=satterth outp=newm;
 random farm(age) rep(farm age) trt*farm(age);
 lsmeans age trt age*trt/cl;
 lsmeans trt/diff adjust=TUKEY;
 lsmeans age*trt/diff slice=age;
 run;

proc print data=newm;
 run;

proc univariate normal plot data=newm;
 var resid;
 run;

proc glimmix data = two;
 where crop = 'm';
 class age farm rep trt;
 model yield  = trt age trt*age/dist=gamma link=log ddfm=satterth;
 random farm(age) rep(farm age) trt*farm(age);
 lsmeans age trt age*trt/cl ilink;
 lsmeans trt/diff adjust=TUKEY ilink;
 lsmeans age*trt/diff slice=age ilink;
 run;
```

2. Example SAS code for analyzing the yield data set in 2007-2008 (L. Zheng and L. Murray)

```
OPTIONS NODATE pageno=1;
data one;
input year $ age farm $ trt $ crop $ rep1yld rep2yld rep3yld @@;
LINES;
2007	1	Naje	2d	d	1055.5058	1141.0853	256.9767
2007	1	Naje	3u	u	1469.186	1780.4651	1865.3876
2007	1	Naje	4d	d	751.4535	1095.7365	188.88075
2007	1	Naje	4u	u	1508.436667	1936.175667	1296.673167
2007	1	Naje	5d	d	737.5	5734.399	225.349
2007	1	Naje	5u	u	1894.38	2096.124	1149.483333
2007	1	Naje	5m	m	400	276	300
2007	1	Naje	6d	d	250.194	250	1751.163
2007	1	Naje	6u	u	301.163	452.965	2777.209
2007	1	Naje	6m	m	250	512.5	162.5
2007	1	Naje	7d	d	318.5755	274.254	819.88375
2007	1	Naje	7u	u	1363.80825	1377.20925	2472.07375
2007	1	Naje	7m	m	250	650	350
2007	1	Naje	8m	m	340	340	120
2007	1.5	Sabang	2d	d	755.814	756.7209	1092.3333
2007	1.5	Sabang	3u	u	1046.7752	970.8837	1065.438
2007	1.5	Sabang	4d	d	634.88375	732.55825	1295.72675
2007	1.5	Sabang	4u	u	1134.257167	1239.341167	1167.907
2007	1.5	Sabang	5d	d	879.7675	523.9825	1085.93
2007	1.5	Sabang	5u	u	1373.953333	247.8683333	556.124
2007	1.5	Sabang	5m	m	430	435	480
2007	1.5	Sabang	6d	d	589.767	1087.636	1169.302
2007	1.5	Sabang	6u	u	1368.527	1581.395	1910.31
2007	1.5	Sabang	6m	m	343.75	375	412.5
2007	1.5	Sabang	7d	d	1004.92725	756.376	1277.3255
2007	1.5	Sabang	7u	u	602.79075	1635.06975	1761.24025
2007	1.5	Sabang	7m	m	550	650	500
2007	1.5	Sabang	8m	m	335	320	310
2007	2	Dorolu	2d	d	382.6357	120.5426	72.7035
2007	2	Dorolu	3u	u	593.0233	119.8643	578.1395
2007	2	Dorolu	4d	d	359.73825	62.03975	72.965
2007	2	Dorolu	4u	u	402.6163333	238.8566667	321.7055
2007	2	Dorolu	5d	d	145.349	48.314	365.5525
2007	2	Dorolu	5u	u	727.3256667	79.45733333	670.6976667
2007	2	Dorolu	5m	m	525.4	551	470.2
```

```
2007    2       Dorolu  6d      d       240.504 199.845 294.186
2007    2       Dorolu  6u      u       290.698 239.147 343.624
2007    2       Dorolu  6m      m       323.5   399.125 427.75
2007    2       Dorolu  7d      d       301.841 60.80425        239.24425
2007    2       Dorolu  7u      u       541.13375       122.19  511.36625
2007    2       Dorolu  7m      m       441.5   605     467.5
2007    2       Dorolu  8m      m       448.4   460     223.3
2008    2       Naje    2d      d       1080.9302       1008.8682       833.8605
2008    2       Naje    3u      u       1174.5155       1480.9109       919.186
2008    2       Naje    4d      d       1078.89525      899.0795        1181.64725
2008    2       Naje    4u      u       881.671 1000.3575       881.671
2008    2       Naje    5d      d       1281.815        1845.814        1127.9975
2008    2       Naje    5u      u       848.4066667     1357.450333     1153.833
2008    2       Naje    5m      m       340     330     240
2008    2       Naje    6d      d       1744.587        1231.473        1641.964
2008    2       Naje    6u      u       1729.432        1831.163        1831.163
2008    2       Naje    6m      m       287.5   362.5   312.5
2008    2       Naje    7d      d       1233.6435       719.62525       771.02725
2008    2       Naje    7u      u       1278.26225      1227.13175      945.914
2008    2       Naje    7m      m       375     400     450
2008    2       Naje    8m      m       320     400     300
2008    2.5     Sabang  2d      d       573.4612        427.5807        578.4916
2008    2.5     Sabang  3u      u       837.4806        725.1357        919.186
2008    2.5     Sabang  4d      d       604.2635        377.66475       478.37525
2008    2.5     Sabang  4u      u       851.3135        612.9456667     715.1033333
2008    2.5     Sabang  5d      d       608.9145        837.258 507.429
2008    2.5     Sabang  5u      u       853.5746667     1195.004333     1177.932667
2008    2.5     Sabang  5m      m       420     380     240
2008    2.5     Sabang  6d      d       661.001 762.694 661.001
2008    2.5     Sabang  6u      u       1524.419        1422.791        1676.86
2008    2.5     Sabang  6m      m       287.5   325     400
2008    2.5     Sabang  7d      d       662.597 611.628 229.3605
2008    2.5     Sabang  7u      u       855.67825       970.62025       881.221
2008    2.5     Sabang  7m      m       300     200     350
2008    2.5     Sabang  8m      m       340     300     330
2008    3       Dorolu  2d      d       767.2661        888.4134        757.1705
2008    3       Dorolu  3u      u       855.5194        1211.9858       1344.3876
2008    3       Dorolu  4d      d       666.00925       452.38375       816.804
2008    3       Dorolu  4u      u       943.1653333     883.6865        679.7588333
2008    3       Dorolu  5d      d       758.527 1719.328        910.2325
2008    3       Dorolu  5u      u       1184.001        1353.144        879.5433333
2008    3       Dorolu  5m      m       460     440     420
2008    3       Dorolu  6d      d       503.23  1006.46 1761.305
2008    3       Dorolu  6u      u       509.819 1223.566        1529.457
2008    3       Dorolu  6m      m       337.5   287.5   450
2008    3       Dorolu  7d      d       1112.36425      606.74425       581.46325
2008    3       Dorolu  7u      u       1195.79775      636.06275       788.71775
2008    3       Dorolu  7m      m       300     400     500
2008    3       Dorolu  8m      m       440     400     880

;

PROC PRINT data=one;
 TITLE 'Yield 2007 and 2008 DATA';
 RUN;

 data two; set one;
 rep = 1; yield =rep1yld; output;
 rep = 2; yield =rep2yld; output;
 rep = 3; yield =rep3yld; output;
 run;
 proc print data=two;
 run;

/*Dinorado data*/
proc mixed  data=two covtest cl;
 where crop = 'd';
 class year farm rep trt;
 model yield = trt year trt*year/ddfm=satterth outp=newd;
 random farm rep(farm) trt*farm trt*rep(farm) year*farm year*rep(farm)
        year*trt*farm;
```

```
 lsmeans year trt year*trt/cl;
 lsmeans trt/diff adjust=TUKEY;
 LSMEANS year*trt/diff slice=year adjust=TUKEY;
 run;

proc print data=newd;
 run;

proc univariate normal plot data=newd;
 var resid;
 run;

/*UPL Ri-5 data*/
proc mixed data=two covtest cl;
 where crop = 'u';
 class year farm rep trt;
 model yield = trt year trt*year/ddfm=satterth outp=newu;
 random farm rep(farm) trt*farm trt*rep(farm) year*farm year*rep(farm)
        year*trt*farm;
 lsmeans year trt year*trt/cl;
 lsmeans trt/diff adjust=TUKEY;
 LSMEANS year*trt/diff slice=year adjust=TUKEY;
 run;
proc univariate normal plot data=newu;
 var resid;
 run;

/*Mungbean data*/
proc mixed data=two covtest cl;
 where crop = 'm';
 class year farm rep trt;
 model yield = trt year trt*year/ddfm=satterth outp=newm;
 random farm rep(farm) trt*farm trt*rep(farm) year*farm year*rep(farm)
        year*trt*farm;
 lsmeans year trt year*trt/cl;
 lsmeans trt/diff adjust=TUKEY;
 LSMEANS year*trt/diff slice=year adjust=TUKEY;
 run;
```

3. R scripts for Figure 2 (L. Zheng)

```
dat1=read.csv("C:/1.Dat2006yield.per.row_formatted.csv")
dat2=read.csv("C:/1.Dat.0708yield.per.row_formatted.csv")
colnames(dat1)
colnames(dat2)
dat = data.frame(rbind(dat1,dat2))

years = c(2006, 2007, 2008)
Dina=c("2d","4d", "5d", "6d","7d")
UPL=c("3u", "4u", "5u", "6u", "7u")
MB=c("8m", "5m","6m","7m")
trts=c(Dina, UPL, MB)

## put all the yield values in a list because the length of yield in 2006,
## 2007 and 2008 differ.

listd=list()
for(i in 1:length(trts)){#length(trts).
   for (j in 1:length(years)){
   dats1=dat[dat$year==years[j]&dat$trt==trts[i],][,6:8]
   datr1 = list(c(apply(dats1,2,as.numeric)))
   listd=c(listd,datr1)
   }
}

listd[[8]][4]<-523
# replaces a big outlier 5734.4 in 5d 2007 using the adjacent value 523.
```

```
p=c(1,4,7,10,13)
b=numeric()
for(i in p){
s=c(i-0.50, i, i+0.50)
b=c(b,s)
}
b

par(mar=c(2.4,4.1,1.8,0.8), oma=c(0,0,3,0))
m <- rbind(1,2,3)
layout(m, width = c(4.6,1.4))
layout.show(3)

plot(seq(0,14,by = (14/(2500 - 1))),1:2500,axes=FALSE, type="n",
xaxt='n',yaxt='n',xlab="",ylab="Yield(g/row)")
axis(2, tck = 1,las=1, col = "grey", lty = "dotted",lwd=0.05)
boxplot(listd[c(1:6,13:15,7:12)], add=TRUE,xlim=c(0,14),boxwex=0.30,
col=c(grey(c(0.12,1.24,2.6)/3)),at = b,medcol=c("white"),axes=FALSE)
box()
axis(side=1,at=p,labels=c("monoculture", "RM", "0.2MB","0.5MB", "0.8MB"))
legend("topleft",c("Dinorado"),cex=1)
legend("top", legend=c("2006","2007","2008"),fill=c(grey(c(0.12,1.24,2.6)/3)),ncol=3, bty="n");

plot(seq(0,14,by = (14/(2800 - 1))),1:2800,axes=FALSE, type="n",
xaxt='n',yaxt='n',xlab="",ylab="Yield(g/row)")
axis(2, tck = 1,las=1, col = "grey", lty = "dotted",lwd=0.05)
boxplot(listd[c(16:21,28:30,22:27)],add=TRUE,xlim=c(0,14),col=c(grey(c(0.12,1.24,2.6)/3)),
boxwex=0.30, at = b, medcol=c("white"), axes=FALSE)
box()
axis(side=1,at=p,labels=c("monoculture", "RM","0.2MB", "0.5MB", "0.8MB"))
legend("topleft",c("UPL Ri-5"),cex=1)

plot(seq(0,14,by = (14/(900 - 1))),1:900,axes=FALSE, type="n",
xaxt='n',yaxt='n',xlab="",ylab="Yield(g/row)")
axis(2, tck = 1,las=1, col = "grey", lty = "dotted",lwd=0.05)
boxplot(listd[c(31:33,40:42,34:39)], add=TRUE,xlim=c(0,14), boxwex=0.30,
col=c(grey(c(0.12,1.24,2.6)/3)),at = b[1:12], axes=FALSE, medcol=c("white"))
box()
axis(side=1,at=p[1:4],labels=c("monoculture","0.2MB","0.5MB", "0.8MB"))
legend("topleft",c("Mung Bean"),cex=1)
```

4. R scripts for Figure 3 (L. Zheng)

```
dat1=read.csv("F:/2.Dat.LER2006_formatted for reps.csv")
dat2=read.csv("F:/2.Dat.LER20078_formatted for reps.csv")
colnames(dat1)
colnames(dat2)
dat = data.frame(rbind(dat1,dat2))

years = c(2006, 2007, 2008) # new variable.
trts=c(4, 5, 6, 7)# new variable.

listd=list()
for(i in 1:length(trts)){#length(trts).
   for (j in 1:length(years)){
   dats1=dat[dat$year==years[j]&dat$trt==trts[i],][,5:7]
   datr1 = list(c(apply(dats1,2,as.numeric)))
   listd=c(listd,datr1)
   }
}

# Reorder the list according to the treatment in Table 2.

listd=listd[c(1:3,10:12,4:9)]

p=c(1,4,7,10) # the centers of box plots for each year.
b=numeric()
for(i in p){
s=c(i-0.50, i, i+0.50)
b=c(b,s)
```

```
}
b


## box plots.

plot(seq(0,13,by = (13/(3.5 - 0))),0:3.5,ylim=c(0,3.5),axes=FALSE, type="n",
xaxt='n',yaxt='n',xlab="",ylab="")
axis(2, tck = 1,las=1, col = "grey", lty = "dotted",lwd=0.05)
boxplot(listd, add=TRUE, xlim=c(0,12),ylim=c(0,3.5),col=c(grey(c(0.12,1.24,2.6)/3)), boxwex=0.30,
at = b, axes=FALSE, medcol=c("white"),ylab="LER", xlab="The mixtures",main=" ")
box()
axis(side=1,at=p,labels=c("RM", "0.2MB","0.5MB", "0.8MB"))
legend("topright",c("2006","2007","2008"),fill=c(grey(c(0.12,1.24,2.6)/3)),cex=0.6)
```

5. R scripts for Figure S3 (L. Zheng)

```
dat1=read.csv("F:/1.Dat2006yield.per.row_formatted.csv")
dat2=read.csv("F:/1.Dat.0708yield.per.row_formatted.csv")
colnames(dat1)
colnames(dat2)
dat = data.frame(rbind(dat1,dat2))
years = c(2006, 2007, 2008)
Dina=c("2d","4d", "5d", "6d","7d")
UPL=c("3u", "4u", "5u", "6u", "7u")
MB=c("8m", "5m","6m","7m")
trts=c(Dina, UPL, MB)
tt=rep(trts,c(rep(3,length(trts))))
year=rep(years,length(trts))

mean.s=numeric()
ses=numeric()
size=numeric()
for(i in 1:length(trts)){#length(trts)
   for (j in 1:length(years)){#length(years)
   dats=dat[dat$year==years[j]&dat$trt==trts[i],][,6:8]
   datr = apply(dats,2,as.numeric)
   means = mean(c(datr),na.rm=T)
   se = sd(c(datr),na.rm=T)
   mean.s=c(mean.s,means)
   ses=c(ses, se)
   size = c(size, length(c(datr))-sum(is.na(datr)))
}
}
mean.s
ses
size

se=ses/sqrt(size)

ll = mean.s-1.96*ses/sqrt(size)
ul = mean.s+1.96*ses/sqrt(size)

mat=data.frame(tt,year, round(cbind(mean.s,se),3), ll, ul)

mat.d=mat[c(1:6,13:15,7:12),]  # CI data for Dinarado.
mat.u=mat[16:30,]  # CI data for UPL.
mat.u=mat.u[c(1:6,13:15,7:12),]
mat.m=mat[-c(1:30),] # CI data for Mung bean.
mat.m=mat.m[c(1:3,10:12,4:9),]

mat.u$tt=rep(c("Monoculture", "RM", "0.2MB","0.5MB","0.8MB"),c(rep(3,5)))
mat.d$tt=rep(c("Monoculture", "RM", "0.2MB","0.5MB","0.8MB"),c(rep(3,5)))
mat.m$tt=rep(c("Monoculture", "0.2MB","0.5MB","0.8MB"),c(rep(3,4)))

mat=rbind(mat.d,mat.u,mat.m)

## 95% CI Plot for yield 2006, 2007 and 2008

x=numeric()
```

```
for(i in 1:5){
xs=c(i-0.15, i, i+0.15)
x=c(x,xs)
}
x

library(plotrix)

par(mar=c(2.4,4.1,1.8,0.8), oma=c(0,0,3,0))
m <- rbind(1,2,3)
layout(m, width = c(4.6,1.4))
layout.show(3)

## plots for Dinorado.
md=matrix(mat.d$mean.s,ncol=5,nrow=3)
rownames(md)=c("2006","2007","2008")
colnames(md)=c("Monoculture","RM","0.2MB","0.5MB","0.8MB")
xs=matrix(x,ncol=5, nrow=3)

suppressWarnings(with(mat.d,
                      plotCI(x,mean.s,2*se,
                             col=as.numeric(year)+20,xlab="", main="",
                             lwd=2,ylab="Mean Yield",
                             ylim=c(min(ll),max(ul)),axes=FALSE)))
box()
axis(side=2,las=1) ## rotate labels for y axis.
axis(side=1,at=1:5,labels=c("monoculture", "RM", "0.2MB","0.5MB", "0.8MB"))  # lables for x axis.
lines(xs[1,],md[1,],type="l",lty=2,col="red")
lines(xs[2,],md[2,],type="l",lty=2,col="green")
lines(xs[3,],md[3,],type="l",lty=2,col="blue")
legend("topright",c("Dinorado"),cex=1)
legend("topleft",
legend=c("2006","2007","2008"),lty=c(1,1,1),col=c("red","green","blue"),ncol=3);

## plots for UPL Ri-5.
ud=matrix(mat.u$mean.s,ncol=5,nrow=3)

suppressWarnings(with(mat.u,
                      plotCI(x,mean.s,2*se,
                             col=as.numeric(year)+20,xlab="",main="",
                             lwd=2,ylab="Mean Yield",
                             ylim=c(min(ll),max(ul)),axes=FALSE)))
box()
axis(side=2,las=1) ## rotate labels
axis(side=1,at=1:5,labels=c("monoculture", "RM", "0.2MB","0.5MB", "0.8MB"))
lines(xs[1,],ud[1,],type="l",lty=2,col="red")
lines(xs[2,],ud[2,],type="l",lty=2,col="green")
lines(xs[3,],ud[3,],type="l",lty=2,col="blue")
legend("topleft",c("UPL Ri-5"),cex=1)

## plots for mung bean.
md=matrix(mat.m$mean.s,ncol=4,nrow=3)
x.m=matrix(x[1:length(mat.m$mean.s)],ncol=4,nrow=3)
suppressWarnings(with(mat.m,
                      plotCI(x[1:length(mean.s)],mean.s,2*se,
                             col=as.numeric(year)+20,xlab="",main="",
                             lwd=2,ylab="Mean Yield",
                             ylim=c(min(ll),max(ul)),axes=FALSE)))

box()
axis(side=2,las=1) ## rotate labels
axis(side=1,at=1:4,labels=c("monoculture", "0.2MB", "0.5MB", "0.8MB"))
lines(x.m[1,],md[1,],type="l",lty=2,col="red")
lines(x.m[2,],md[2,],type="l",lty=2,col="green")
lines(x.m[3,],md[3,],type="l",lty=2,col="blue")
legend("topright",c("Mung Bean"),cex=1)
```

6. R scripts for Figure S4 (L. Zheng)

```
dat1=read.csv("F:/2.Dat.LER2006_formatted for reps.csv")
```

```
dat2=read.csv("F:/2.Dat.LER20078_formatted for reps.csv")
colnames(dat1)
colnames(dat2)
dat = data.frame(rbind(dat1,dat2))

years = c(2006, 2007, 2008) # new variable.
trts=c(4, 5, 6, 7)# new variable.
tt=rep(trts,c(rep(3,length(trts))))
year=rep(years,length(trts))

mean.s=numeric()
ses=numeric()
size=numeric()
for(i in 1:length(trts)){#length(trts)
   for (j in 1:length(years)){#length(years)
   dats=dat[dat$year==years[j]&dat$trt==trts[i],][,5:7]
   datr = apply(dats,2,as.numeric)
   means = mean(c(datr),na.rm=T)
   se = sd(c(datr),na.rm=T)
   mean.s=c(mean.s,means)
   ses=c(ses, se)
   size = c(size, length(c(datr))-sum(is.na(datr)))
}
}
mean.s
ses
size

se=ses/sqrt(size)

ll = mean.s-1.96*ses/sqrt(size)
ul = mean.s+1.96*ses/sqrt(size)


tt=rep(c("RM","0.2MB","0.5MB","0.8MB"),c(rep(3,length(trts))))
mat=data.frame(tt,year, round(cbind(mean.s[c(1:3,10:12,4:9)],se),3), ll, ul)
colnames(mat)=c("Treatment","Year","Mean","SE","LL","UL")

## Make 95% CI Plot for LER 2006, 2007 and 2008


x=numeric()  # x is the vector of location for each CI plot.
for(i in 1:4){
xs=c(i-0.15, i, i+0.15)
x=c(x,xs)
}
x

library(plotrix)

## CI plots.

plot(seq(0.5,4.5,by = (4/(2 - 0))),0:2,ylim=c(0.6,2),axes=FALSE, type="n",
xaxt='n',yaxt='n',xlab="The mixtures",ylab="LER")
axis(2, tck =1,las=1, col = "grey", lty = "dotted",lwd=0.05)

suppressWarnings(with(mat,
                      plotCI(x,mat$Mean,2*mat$SE,
                             col=as.numeric(year)+20,, main="",
                             lwd=2,ylab="",
                             ylim=c(min(mat$LL),max(mat$UL)),add=TRUE,axes=FALSE)))

box()
axis(side=2,las=1) ## rotate labels for y axis.
axis(side=1,at=1:4,labels=c("RM", "0.2MB","0.5MB", "0.8MB"))  # labels for x axis.
legend("topright",c("2006","2007","2008"),lty=c(1,1,1),col=c("red","green","blue"),cex=.6)
```
